# A general model for head and neck auto‐segmentation with patient pre‐treatment imaging during adaptive radiation therapy

**DOI:** 10.1002/mp.17732

**Published:** 2025-03-07

**Authors:** Brett Clark, Nicholas Hardcastle, Mathieu Gaudreault, Leigh A. Johnston, James C. Korte

**Affiliations:** ^1^ Department of Biomedical Engineering University of Melbourne Melbourne Australia; ^2^ Department of Physical Sciences Peter MacCallum Cancer Centre Melbourne Australia; ^3^ Centre for Medical Radiation Physics University of Wollongong Wollongong Australia; ^4^ Sir Peter MacCallum Department of Oncology University of Melbourne Melbourne Australia; ^5^ Melbourne Brain Centre Imaging Unit University of Melbourne Melbourne Australia; ^6^ Graeme Clark Institute University of Melbourne Melbourne Australia

**Keywords:** adaptive radiation therapy, head and neck, image segmentation

## Abstract

**Background:**

During head and neck (HN) radiation therapy, patients may undergo anatomical changes due to tumor shrinkage or weight loss. For these patients, adaptive radiation therapy (ART) is required to correct treatment plans and to ensure that the prescribed radiation dose is delivered to the tumor while minimizing dose to the surrounding organs‐at‐risk (OARs). Patient pre‐treatment images and segmentation labels are always available during ART and may be incorporated into deep learning (DL) auto‐segmentation models to improve performance on mid‐treatment images.

**Purpose:**

Existing DL methods typically incorporate pre‐treatment data during training. In this work, we investigated whether including pre‐treatment data at inference time would affect model performance, as inference‐time inclusion would eliminate the requirement for costly model retraining for new patient cohorts.

**Methods:**

We developed a general adaptive model (GAM) that included pre‐treatment data at inference time through additional input channels. We compared the GAM with a patient‐specific model (PSM), which included pre‐treatment data during training, a reference model (RM), which did not include pre‐treatment data, and a rigid image registration (RIR) method. Models were developed using a large dataset of pre‐ and mid‐treatment computed tomography images and segmentation labels (primary gross tumor volume [GTVp] and 16 OARs) for 110 patients who underwent ART for HN cancer.

**Results:**

The GAM showed improved performance over the PSM and RM for several structures, with the largest differences in dice similarity coefficient for difficult‐to‐segment structures: the GTVp (RM: 0.17, PSM: 0.36, GAM: 0.61, RR: 0.65) and left/right brachial plexus (RM: 0.38/0.35, PSM: 0.43/0.43, GAM: 0.49/0.49, RR: 0.36/0.38). The GAM attained similar performance to RR for all structures except the brainstem (GAM: 0.82, RR: 0.74), mandible (GAM: 0.88, RR: 0.68), and spinal cord (GAM: 0.76, RR: 0.51), for which the GAM performed higher.

**Conclusion:**

The inclusion of patient pre‐treatment images and segmentation labels can improve auto‐segmentation performance during HN ART, in particular for structures with high variability or low contrast. Including pre‐treatment data at DL model inference time (GAM) may give improvements over standard DL models for the GTVp and several OARs, while eliminating the need for costly model retraining with new patient cohorts. However, rigid registration provides similar performance to adaptive DL models for the GTVp and most OARs.

## 1 INTRODUCTION

Patients undergoing radiation therapy (RT) for head and neck (HN) cancer often experience anatomical changes due to weight loss, tumor shrinkage, or organ‐at‐risk (OAR) volume and shape changes.^1^ Adaptive radiation therapy (ART) allows for revision of treatment plans in response to changing anatomy, and is essential in ensuring delivery of the prescribed radiation dose to tumor volumes while minimizing dose to normal tissue.^2^, ^3^ However, manual components of the ART workflow, such as delineating (segmenting) the tumor and organs‐at‐risk (OARs) on computed tomography (CT) images, are time‐consuming. Indeed, manual segmentation is the largest barrier to the increased adoption of ART.^4^

Auto‐segmentation is the application of algorithms to the problem of segmenting anatomical structures and can be applied to reduce segmentation times and improve treatment consistency. While traditional auto‐segmentation techniques used image intensities alone (e.g., thresholding,^5^ region‐growing^6^), later methods incorporated prior knowledge through manual segmentation labels (e.g., atlas methods,^7^ active shape,^8^ and appearance^9^ models). Recently, deep learning (DL) methods, trained on large labeled segmentation datasets, have shown state‐of‐the‐art performance for many segmentation tasks.^10^ Most DL auto‐segmentation models employ some variant of the U‐Net architecture,^11^ including all entries into a recent HN segmentation challenge.^12^ When performing auto‐segmentation during replanning for ART, rigid (RIR) or deformable image registration (DIR) contour propagation methods are commonly used. However, DL methods have recently shown similar performance to DIR methods for HN auto‐segmentation, while being faster to compute.^13^

During ART, patient pre‐treatment imaging and segmentation labels (pre‐treatment data) are available and may improve auto‐segmentation performance on mid‐treatment imaging of the same patient. Previous DL approaches have included pre‐treatment data at training time to improve auto‐segmentation performance, using either fine‐tuning^14^, ^15^ or pooling^16^, ^17^ methods. However, these methods require an additional model to be trained for each new patient, a time‐consuming task that can take approximately 56 h^18^ and may be prohibitive to adaptive treatment. Works that incorporated pre‐treatment data at inference time are mostly limited to combined image registration and segmentation methods.^19^, ^20^ Existing HN work^21^ has explored the use of pre‐treatment data at inference time; however, this work used DIR as a preprocessing step, and therefore could not attain the fast inference times possible with DL‐only approaches. Also, due to the small size of their adaptive dataset (nine patients), this work required an additional DL model for inference, trained on a large non‐adaptive dataset.

In this work, we collected a large dataset of paired pre‐ and mid‐treatment CT images and segmentation labels for 110 patients. To the best of our knowledge, this is the largest such dataset for HN auto‐segmentation during ART. We developed a general adaptive model (GAM) that included patient pre‐treatment data at inference time, thereby eliminating the need to perform costly model retraining for each new patient cohort. We compared the performance of the GAM with that of a patient‐specific model (PSM) that included patient pre‐treatment data at training time via pooling, a reference model (RM) that included no patient pre‐treatment data, and a pre‐treatment label propagation method using RIR.

## 2 MATERIALS

### 2.1 PMCC‐REPLAN dataset

This dataset consisted of both pre‐ and mid‐treatment CT images with tumor and OAR segmentation labels for 110 patients (220 CT images) who underwent ART during treatment for HN cancer at the Peter MacCallum Cancer Centre (PMCC) (Australia, 2018–2022). The median time between pre‐ and mid‐treatment images was 36 days (range: 14–79 days) (see Table S3). Patients were imaged using Philips Brilliance Big Bore CT scanners (140 kVp) with a median field‐of‐view of 600 × 600 × 457  mm and voxel spacing of 1.17 × 1.17 × 2.00 mm. Iodinated intravenous contrast was administered in 82.0% of pre‐treatment and 17.7% of mid‐treatment cases. Approval to conduct this retrospective study was given by the PMCC ethics committee.

The primary gross tumor volume (GTVp) and 16 OARs (left/right brachial plexus, brain, brainstem, esophagus, larynx, left/right lens, mandible, oral cavity, left/right parotid, pharyngeal constrictors, spinal cord, and left/right submandibular) were manually segmented by radiation therapists and radiation oncologists during treatment planning.

The PMCC‐REPLAN dataset had imbalanced segmentation label numbers (class imbalance) with a range of 64–215 labels per OAR (see Table S1). OAR volumes were also highly imbalanced, with the brain (mean volume: 1400 cm^3^) having a mean volume approximately 4500 times larger than the left/right lens (mean volume: 0.31 cm^3^) (see Table S2).

### 2.2 Data preprocessing

Preprocessing of OAR labels was performed to interpolate missing slices, followed by connected component analysis to remove disconnected foreground voxels. Pre‐treatment images were aligned with mid‐treatment images using RIR (SimpleITK library,^22^ v2.2.1, Python). RIR was performed at increasing resolutions (downsampled by factors of 4, 2, and 1) using a mutual information loss function with stochastic gradient descent optimizer and a learning rate of 1. CT images and GTVp/OAR labels were resampled to 1 × 1 × 2  mm and cropped to 330 × 400 × 500  mm surrounding the HN area to reduce the graphics processing unit (GPU) memory usage. The HN area was localized using a public brain segmentation model.^23^

## 3 METHODS

### 3.1 Auto‐segmentation models

**FIGURE 1:**
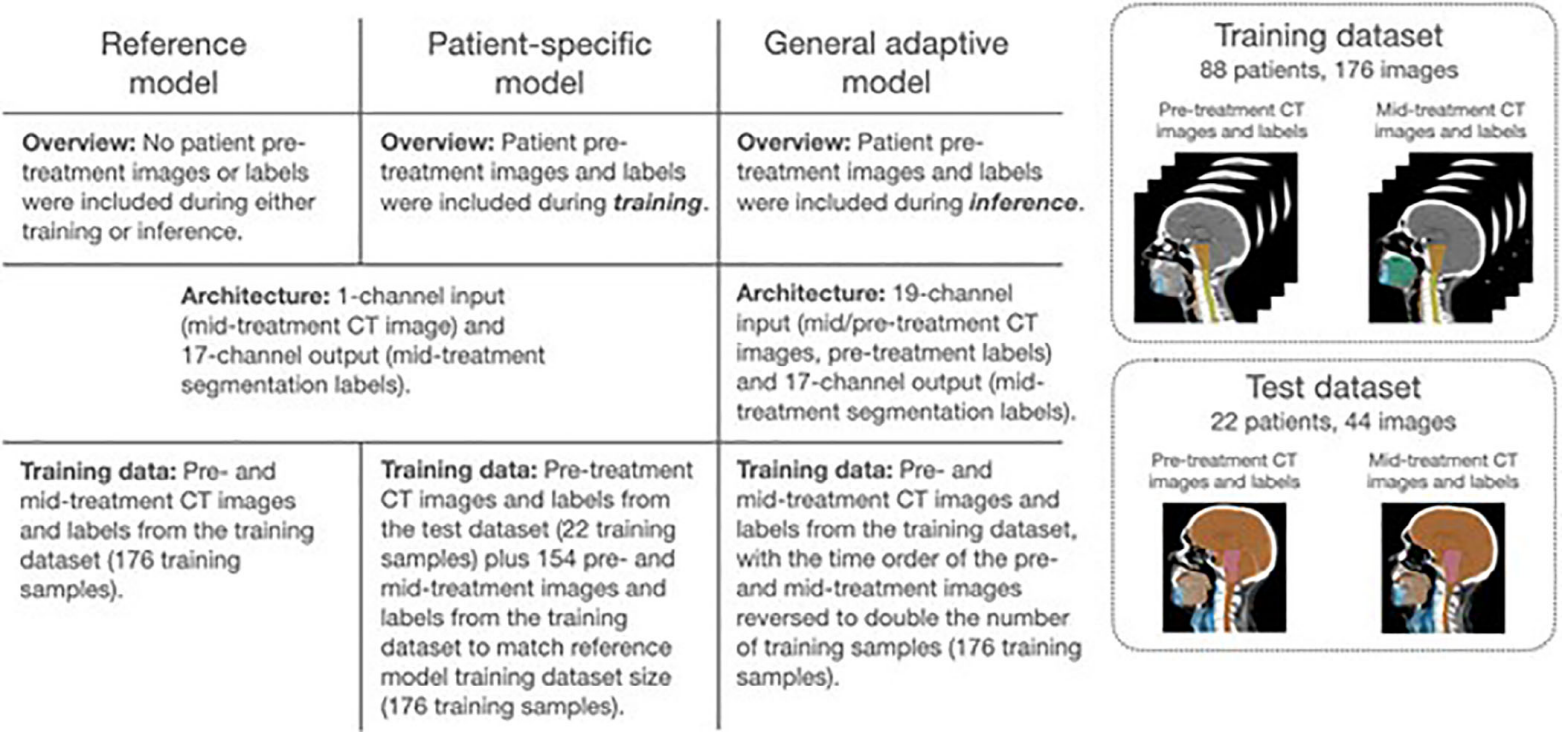
An overview of the reference, patient‐specific, and general adaptive models and the PMCC‐REPLAN dataset, on which they were trained and evaluated. Model differences are outlined, including architecture and training data.

Three DL auto‐segmentation models were trained to segment 17 structures in the HN, with each model using a different method to incorporate patient pre‐treatment data (see Figure 1). The RM, which did not incorporate pre‐treatment data during either training or inference, was trained as a baseline from which to evaluate subsequent models. The PSM included pre‐treatment data from all 22 test set patients by incorporating it into the training dataset. The GAM incorporated pre‐treatment data at inference time through additional model input channels. The GAM input was modified to accept the mid‐treatment CT image, plus the pre‐treatment CT image and GTVp/OAR labels (19 input channels), whereas both RM and PSM models accepted only the mid‐treatment CT image as input. All models used a 3D U‐Net architecture with four down/upsampling layers, instance normalization, and ReLU activations^24^ (see Figure S1).

To validate our model architecture and training process, we compared the RM performance with that of a model trained using the popular nnU‐Net framework^25^ (v2.5.1). For the nnU‐Net model, a high‐resolution 3D U‐Net with residual encoder was used without model ensembling and without access to pre‐treatment data. When performing multi‐structure segmentation with nnU‐Net, ground truth segmentation labels must be present for each structure for each training patient. As PMCC‐REPLAN contained patients with missing labels, we performed single‐structure training with nnU‐Net for each of the 17 structures in the dataset. Additionally, we compared our models that incorporated pre‐treatment data (PSM and GAM) with another method: pre‐treatment label propagation via RIR. Multi‐resolution RR was performed with SimpleITK using the same parameters described in the data preprocessing section.

### 3.2 Training data

Models were trained and evaluated using five‐fold cross‐validation on the PMCC‐REPLAN dataset. The dataset was split, at the patient level, into five equally sized folds, with each fold containing pre‐ and mid‐treatment CT images and GTVp/OAR labels for 22 patients. For each of the five cross‐validation runs, models were presented with 70 training, 18 validation, and 22 test patients.

Training datasets differed between RM, PSM, and GAM models due to the inclusion or exclusion of patient pre‐treatment data, and the method of inclusion. The RM was trained using all pre‐ and mid‐treatment images from the training dataset (176 training samples), without access to any test fold patients’ pre‐treatment data. The PSM was trained on the same data as the RM but also included pre‐treatment images from the test dataset (22 training samples), thereby including patient pre‐treatment data during training. PSM training sample numbers were matched with the RM by removing 22 other samples from the training dataset. The GAM was also trained on all training dataset images. When training the GAM, the time order of the pre‐ and mid‐treatment images was also reversed, to double the number of training samples and to ensure that all labels were used at both input and output (176 training samples).

### 3.3 Model training

Models were trained for 1000 epochs using a hybrid dice with focal loss.^26^ Due to class imbalance, individual OAR contributions to the loss function were weighted by their inverse frequency in the training dataset.^27^ Model parameters were updated using the Adam optimizer^28^ with a learning rate of 0.001. To reduce overfitting, data augmentation was applied in the form of random translation (± 50 mm), rotation (± 5 degrees), and scaling (0.8 – 1.2 x) using the TorchIO library (v0.18.14).^29^ The validation dataset was used to select the final model parameters.

Two methods were used to improve segmentation for small OARs, which are typically difficult to segment in a multi‐organ setting due to the large volume imbalance between OARs. These methods were using a dice‐based loss function to minimize the class‐level error and applying individual weightings to each OAR term in the loss function. OAR weightings were calculated using the inverse of the OAR volume across the training dataset. To encourage convergence (non‐zero validation DSC) of the small OARs, while not unduly penalising large OARs, these weightings were only applied for the first 200 training epochs.

Models were implemented in Python (v3.10.4) using the PyTorch^30^ library (v2.0.1) and PyTorch Lightning.^31^ Training was performed using mixed precision (bfloat16) data types on an NVIDIA A100 (80GB) GPU with CUDA Toolkit (v11.7). The source code is available at https://github.com/clarkbab/hn‐general‐adaptive‐model.

### 3.4 Model evaluation

Models were evaluated using the dice similarity coefficient (DSC),^32^ a measure of label overlap, and the mean surface distance (MSD),^33^ a measure of the average distance between label boundaries. All predictions were resampled to the original CT image resolution before evaluation. Significant differences in model performance were calculated using the corrected resampled *t*‐test^34^ that accounts for reduced test score variance due to training dataset overlap when performing five‐fold cross‐validation.

## 4 RESULTS

### 4.1 Model convergence

Of the 15 models developed in this work (three models, five folds), 3 achieved convergence for the GTVp and all 16 OARs and the remainder achieved convergence for the GTVp and 15 OARs each (see Table S3). For the models that did not achieve convergence for all structures, the left or right lens did not converge for nine runs, and the right brachial plexus, brainstem, and left submandibular did not converge for one run each.

### 4.2 Deep learning model performance

**FIGURE 2:**
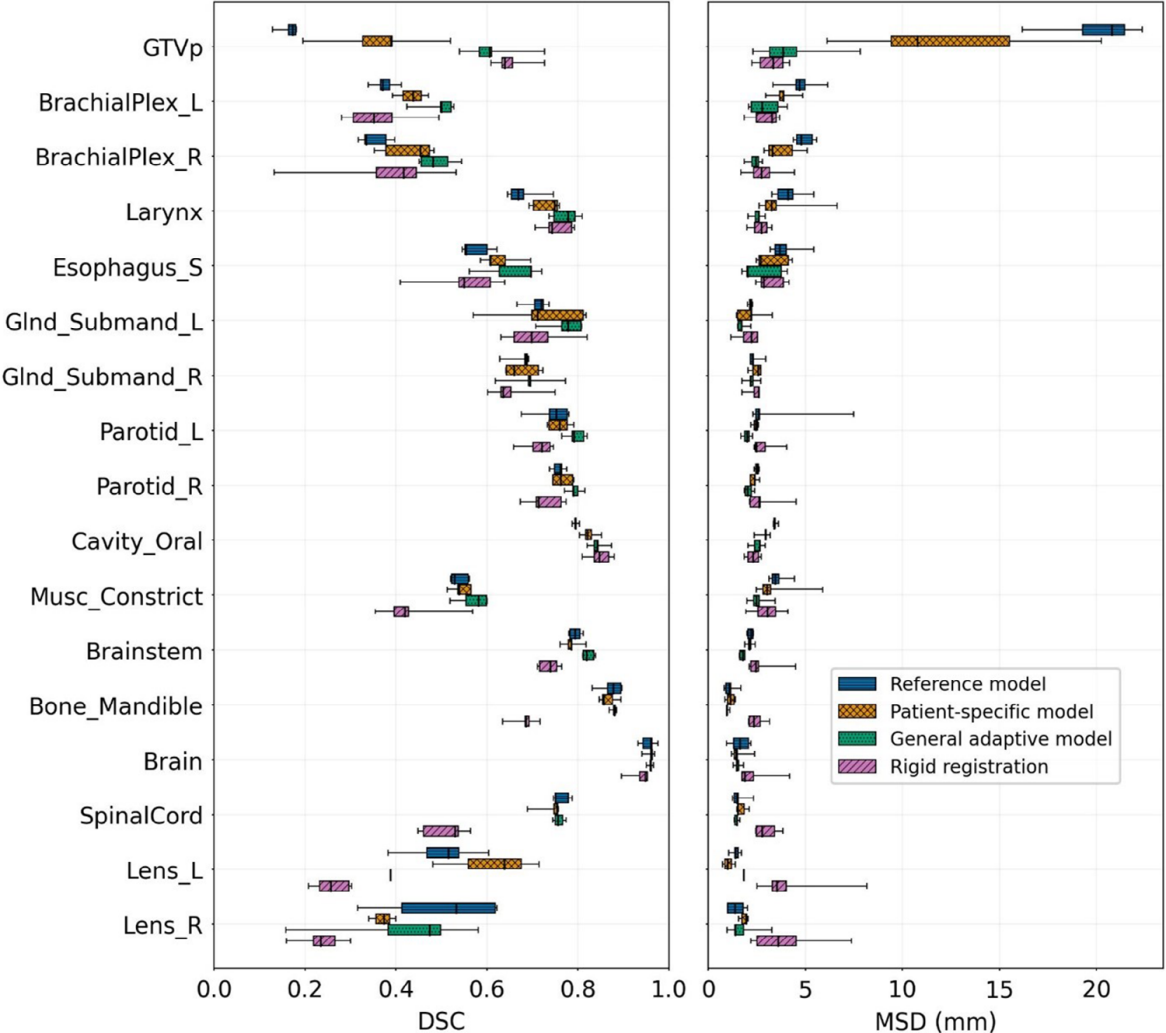
Mean DSC and MSD (mm) for reference, patient‐specific, general adaptive, and rigid image registration methods trained and evaluated on the PMCC‐REPLAN dataset. Mean DSC and MSD are shown per test fold (box plots) for the GTVp and 16 OARs, with rows ordered by largest DSC differences between general adaptive and reference models.

When comparing the performance of the DL models trained in this work, the GAM attained a significantly higher (*p* < 0.05) mean DSC than the RM for the GTVp and seven OARs, with largest differences for the GTVp (0.45), left/right brachial plexus (0.12/0.14), larynx (0.09), left submandibular (0.06), and oral cavity (0.05) (see Table 1, Figure 2). The GAM achieved a higher mean DSC than the PSM for the GTVp and three OARs with largest differences for the GTVp (0.25), right brachial plexus (0.06), and the right parotid (0.03). When considering mean MSD, the GAM showed a significant improvement over the RM for the GTVp and six OARs, with largest differences for the GTVp (15.68 mm), right brachial plexus (2.54 mm), esophagus (1.25 mm), oral cavity (0.90 mm), and brainstem (0.43 mm), and showed significantly better performance than the PSM for three OARs (right brachial plexus, brainstem, and esophagus) (see Table S4).

**TABLE 1:** DSC (mean ± std. deviation) for reference (RM), patient‐specific (PSM), general adaptive (GAM), and rigid image registration (RIR) auto‐segmentation methods, trained and evaluated on the PMCC‐REPLAN dataset.

Structure	DSC			
	Reference	Patient‐specific	General adaptive	Rigid registration
GTVp	0.17 ± 0.02	* 0.36 ± 0.12	*† 0.61 ± 0.07	0.65 ± 0.04
BrachialPlex_L	0.38 ± 0.03	0.43 ± 0.03	* 0.49 ± 0.04	0.36 ± 0.08
BrachialPlex_R	0.35 ± 0.03	0.43 ± 0.06	*† 0.49 ± 0.04	0.38 ± 0.15
Larynx	0.68 ± 0.04	0.73 ± 0.03	* 0.77 ± 0.03	0.75 ± 0.04
Esophagus_S	0.57 ± 0.03	0.63 ± 0.04	0.66 ± 0.07	0.55 ± 0.09
Glnd_Submand_L	0.71 ± 0.03	0.72 ± 0.10	* 0.77 ± 0.04	0.71 ± 0.07
Glnd_Submand_R	0.68 ± 0.03	0.68 ± 0.04	0.69 ± 0.05	0.65 ± 0.06
Parotid_L	0.74 ± 0.04	0.76 ± 0.03	0.80 ± 0.02	0.71 ± 0.04
Parotid_R	0.76 ± 0.01	0.77 ± 0.02	*† 0.79 ± 0.02	0.73 ± 0.04
Cavity_Oral	0.80 ± 0.01	0.82 ± 0.02	* 0.84 ± 0.02	0.85 ± 0.03
Musc_Constrict	0.54 ± 0.02	0.54 ± 0.02	0.57 ± 0.03	0.43 ± 0.08
Brainstem	0.79 ± 0.02	0.79 ± 0.02	*†‡ 0.82 ± 0.01	0.74 ± 0.02
Bone_Mandible	0.87 ± 0.03	0.87 ± 0.02	‡ 0.88 ± 0.01	0.68 ± 0.03
Brain	0.95 ± 0.02	0.96 ± 0.01	0.96 ± 0.01	0.94 ± 0.02
SpinalCord	0.76 ± 0.02	0.74 ± 0.03	‡ 0.76 ± 0.01	0.51 ± 0.05
Lens_L	0.50 ± 0.08	0.61 ± 0.12	0.39	0.26 ± 0.04
Lens_R	0.50 ± 0.15	0.37 ± 0.03	0.42 ± 0.16	0.24 ± 0.05
All structures	0.63 ± 0.20	0.66 ± 0.17	0.69 ± 0.16	0.60 ± 0.20

Mean DSC is averaged over the five test folds for the GTVp and 16 OARs, with rows ordered by largest difference between GAM and RM methods. Significant improvements (*p* < 0.05) in GAM performance over RM (^*^) and PSM (^†^) methods are shown, in addition to significant differences between GAM and RR (^‡^). No std. deviation was available for the GAM for the left lens due to convergence for a single test fold only.

The PSM achieved significantly higher mean DSC than the RM for the GTVp with a difference of 0.20. For the mean MSD, the PSM showed no significant improvement in performance over the RM for the GTVp or any OAR.

The RM did not achieve significantly higher performance than either GAM or PSM models for the GTVp or any OAR using any metric.

### 4.3 Rigid registration performance

When comparing the GAM and PSM models with RIR, the GAM attained significantly higher mean DSC than RIR for the brainstem, mandible, and spinal cord (differences: 0.08, 0.20, 0.25 respectively) (see Table 1, Figure 2). Additionally, the PSM attained higher mean DSC than RIR for the same three OARs plus the right lens with respective differences of 0.05, 0.19, 0.23, and 0.13. RIR showed no mean DSC improvement over the GAM and improvement over the PSM for the GTVp alone (difference: 0.29).

### 4.4 nnU‐Net performance

When validating the performance of our RM model against the nnU‐Net framework, nnU‐Net achieved significantly higher mean DSC for three OARs, the esophagus, right parotid, and right submandibular, with differences of 0.06, 0.04, and 0.08, respectively (see Table S6). When using mean MSD, nnU‐Net showed significant improvements over the RM for the right parotid and right submandibular (respective differences: 0.54 , 0.70 mm).

## 5 DISCUSSION

The improved performance of models that incorporated patient pre‐treatment data demonstrated the benefit of using this data for auto‐segmentation during adaptive HN RT. Both the GAM and PSM models showed improved performance over the RM when segmenting the GTVp. Additionally, the GAM showed superior performance to the RM for seven OARs.

The GAM was superior to the PSM for the GTVp and three OARs, indicating that pre‐treatment data can be included at inference time, via additional input channels, rather than during training. This eliminates the need for costly retraining of models for each new patient treated using ART. Existing work^21^ found improvements for seven OARs (brainstem, larynx, oral cavity, parotid glands, submandibular glands) when including pre‐treatment data during inference for HN auto‐segmentation. However, this work incorporated DIR as a preprocessing step, thereby increasing inference times, as DIR auto‐segmentation methods can take approximately seven times longer than DL methods.^13^ Also, this work required an additional model for inference, trained on a large non‐adaptive segmentation dataset, due to the small size of their adaptive dataset (nine patients). The differences in performance that we observed between GAM and PSM models are not to be expected necessarily, as both models incorporated the same pre‐treatment data, albeit using different methods. In our work, the PSM included patient pre‐treatment data during training, in the same manner as other training samples, whereas alternative strategies might emphasize pre‐treatment data through oversampling or fine‐tuning.^13^

When comparing the GAM with pre‐treatment label propagation using RIR, the GAM showed improvements over RIR for several OARs. Large improvements were seen for the mandible and spinal cord. DL models typically attain good segmentation scores for these high‐contrast structures, while RIR methods may struggle due to local, non‐rigid movement of the head and spine between pre‐ and mid‐treatment images.

Similarly to a previous fine‐tuning study,^35^ we observed a large difference in GTVp segmentation performance between DL models that included pre‐treatment data and those that did not. This difference emphasizes the inability of standard DL models to generalize well to new patients given the variability in GTVp shape, size, and location (see Figure S1). In comparison, RIR performed as well as the best DL model (GAM) for GTVp segmentation, showing that pre‐treatment data can be incorporated using either DL or registration to improve mid‐treatment segmentation. However, the reliance of these methods on pre‐treatment data makes them susceptible to the propagation of errors present in this data.

The GAM showed a large improvement in performance over the RM for the left and right brachial plexus. These low‐contrast OARs are typically difficult to segment on CT images and pose a challenge for DL auto‐segmentation models. RIR attained similar performance to the GAM for the brachial plexus, emphasizing the utility of pre‐treatment data when segmenting these structures. For high‐contrast OARs (brain, mandible, spinal cord), the GAM showed no performance advantages over the RM. Additionally, RIR performed very poorly for two of these structures (mandible, spinal cord), suggesting that pre‐treatment data is of limited utility when OAR boundaries are clearly defined. As a result, improved image contrast, using magnetic resonance (MR) imaging, for example, could reduce the need for pre‐treatment data. Indeed, for the brachial plexus^36^ and some primary tumors (e.g., of the oral cavity and oropharynx^37^), it is recommended to include MR imaging, in addition to CT, for improved boundary contrast. The PMCC‐REPLAN dataset did not incorporate MR images, as these are not typically acquired during mid‐treatment replanning at our center. However, future work could investigate the use of synthetic MR generation models,^38^ trained on large datasets of unpaired CT and MR images, to improve segmentation performance for these structures.

The popular nnU‐Net framework was used to validate our RM model performance. While improvements were seen when using nnU‐Net in place of the RM for some OARs, no performance differences were seen for the GTVp or brachial plexus, which showed the largest differences between GAM and RM models. These results indicate that improvements observed for GAM models for these structures were due to the inclusion of pre‐treatment data, and not due to RM model architecture or training protocol deficiencies. Additionally, nnU‐Net performance may be somewhat inflated, as these models were single‐structure models in comparison to the 17‐structure models used by the RM. To the best of our knowledge, it is not currently possible to train an nnU‐Net multi‐structure model without a complete set of segmentation labels for all structures for all patients (https://github.com/MIC‐DKFZ/nnUNet/issues/2517).

## 6 CONCLUSION

The inclusion of patient pre‐treatment images and segmentation labels can improve auto‐segmentation performance during HN ART, in particular for structures with high variability or low contrast. Including pre‐treatment data at DL model inference time (GAM) may give improvements over standard DL models for the GTVp and several OARs, while eliminating the need for costly model retraining with new patient cohorts. However, RIR provides similar performance to adaptive DL models for the GTVp and most OARs.

## CONFLICT OF INTEREST STATEMENT

Nicholas Hardcastle receives research grant support from Varian Medical Systems and Reflexion Medical for unrelated research. He is a paid consultant of SeeTreat Medical.

## Supporting information



Supplementary Figure 1: 3D U‐Net model architecture. Reference (RM) and patient‐specific (PSM) models accept single‐channel inputs (mid‐treatment image, C = 1). General adaptive model (GAM) takes pre/mid‐treatment CT plus pre‐treatment labels (17 structures) as input (C = 19). Conv block (blue): 3D convolution (kernel: 3, stride: 1, padding: 1), instance normalization, and rectified linear unit activation (ReLU) layers. Down block (orange): max pooling (kernel: 2, stride: 2, padding: 0). Up block: transposed convolution (kernel: 2, stride: 2, padding: 0).

Supplementary Figure 2: Example reference model (RM) and general adaptive model (GAM) predictions (orange) when segmenting the GTVp on a mid‐treatment CT image. Mid‐ and pre‐treatment GTVp ground truth labels (blue) are shown for reference.

Supplementary Table 1: GTVp and OAR segmentation label numbers for the PMCC‐REPLAN dataset (110 patients, 220 CT images) split by pre‐treatment, mid‐treatment, and total label numbers.

Supplementary Table 2: Mean volumes (cm^3^) for the GTVp and OARs in the PMCC‐REPLAN dataset (110 patients, 220 CT images) split by pre‐ and mid‐treatment images. Significant differences (*) in volume between pre‐ and mid‐treatment images were calculated using a paired *t*‐test (*p* < 0.05).

Supplementary Table 3: Patient demographics for PMCC‐REPLAN dataset.

Supplementary Table 4: OARs that failed to converge (non‐zero validation DSC) for a given model and test fold.

Supplementary Table 5: MSD (mean ± std. deviation) for reference (RM), patient‐specific (PSM), general adaptive (GAM), and rigid image registration (RIR) auto‐segmentation methods, trained and evaluated on the PMCC‐REPLAN dataset. Mean DSC is averaged over the five test folds for the GTVp and 16 OARs, with rows ordered by largest difference between GAM and RM methods. Significant improvements (*p* < 0.05) in GAM performance over RM (*) and PSM (†) methods are shown, in addition to significant differences between GAM and RR (‡). No std. deviation was available for the GAM for the left lens due to convergence for a single test fold only.

Supplementary Table 6: DSC (mean ± std. deviation) and MSD (mean ± std. deviation) for reference and nnU‐Net models trained and evaluated on the PMCC‐REPLAN dataset. Mean DSC and MSD are averaged over all test folds for the GTVp and 16 OARs. Significant improvements in performance (*p* < 0.05) are shown for reference (*) and nnU‐Net (†) models.
