# Supplementary figures and images for: A general model for head and neck auto‐segmentation with patient pre‐treatment imaging during adaptive radiation therapy
Source: Med Phys. 2025 Mar 7;52(6):4590–7. doi: 10.1002/mp.17732 (PMC12149676; doi:10.1002/mp.17732)

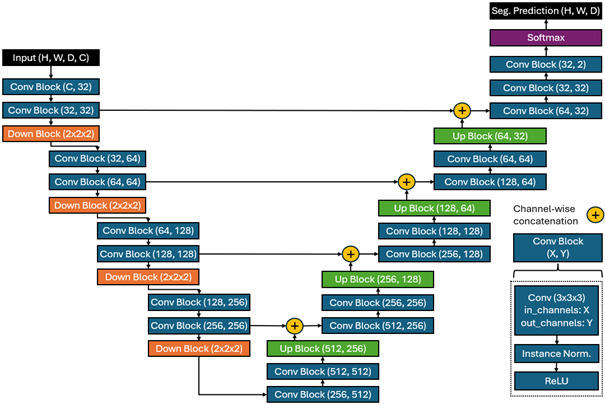

Supplement: Supplementary file 1 — Supplementary Figure 1: 3D U‐Net model architecture. Reference (RM) and patient‐specific (PSM) models accept single‐channel inputs (mid‐treatment image, C = 1). General adaptive model (GAM) takes pre/mid‐treatment CT plus pre‐treatment labels (17 structures) as input (C = 19). Conv block (blue): 3D convolution (kernel: 3, stride: 1, padding: 1), instance normalization, and rectified linear unit activation (ReLU) layers. Down block (orange): max pooling (kernel: 2, stride: 2, padding: 0). Up block: transposed convolution (kernel: 2, stride: 2, padding: 0). [file MP-52-4590-s006.jpg]

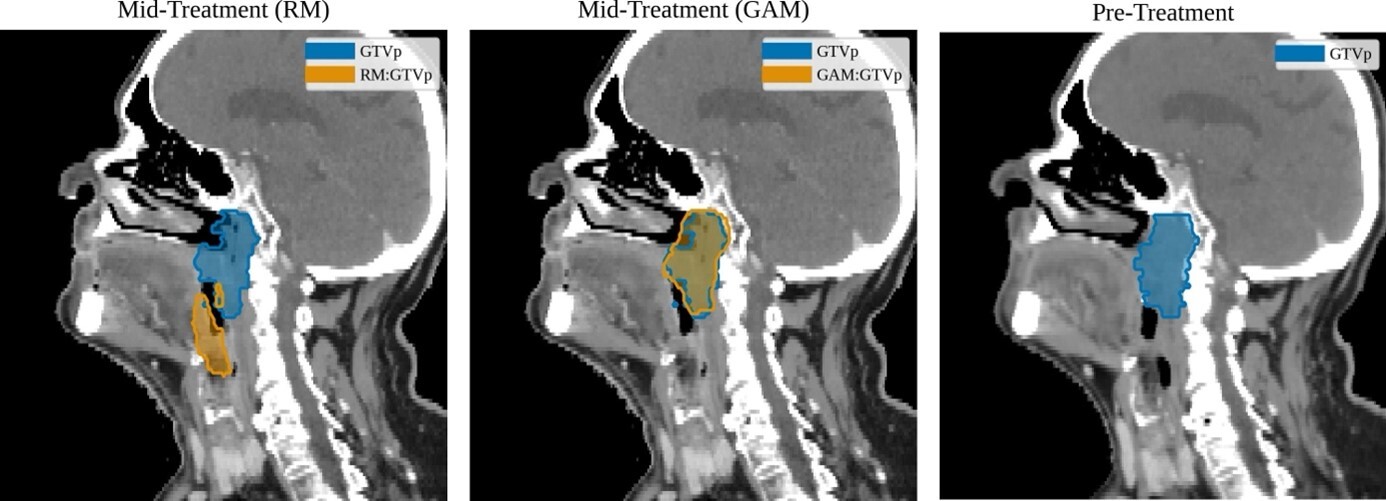

Supplement: Supplementary file 2 — Supplementary Figure 2: Example reference model (RM) and general adaptive model (GAM) predictions (orange) when segmenting the GTVp on a mid‐treatment CT image. Mid‐ and pre‐treatment GTVp ground truth labels (blue) are shown for reference. [file MP-52-4590-s003.jpg]
