# Supplementary material for: A general model for head and neck auto‐segmentation with patient pre‐treatment imaging during adaptive radiation therapy
Source: Med Phys. 2025 Mar 7;52(6):4590–7. doi: 10.1002/mp.17732 (PMC12149676; doi:10.1002/mp.17732)
Supplement: Supplementary file 3 — Supplementary Table 1: GTVp and OAR segmentation label numbers for the PMCC‐REPLAN dataset (110 patients, 220 CT images) split by pre‐treatment, mid‐treatment, and total label numbers. [file MP-52-4590-s008.docx]

| Structure | Label numbers | | |
| --- | --- | --- | --- |
|  | Pre-treatment | Mid-treatment | Total |
| Bone_Mandible | 107 | 108 | 215 |
| BrachialPlex_L | 80 | 78 | 158 |
| BrachialPlex_R | 76 | 72 | 148 |
| Brain | 74 | 77 | 151 |
| Brainstem | 101 | 101 | 202 |
| Cavity_Oral | 79 | 83 | 162 |
| Esophagus_S | 77 | 76 | 153 |
| GTVp | 101 | 99 | 200 |
| Glnd_Submand_L | 37 | 27 | 64 |
| Glnd_Submand_R | 37 | 33 | 70 |
| Larynx | 69 | 68 | 137 |
| Lens_L | 37 | 32 | 69 |
| Lens_R | 37 | 30 | 67 |
| Musc_Constrict | 99 | 99 | 198 |
| Parotid_L | 97 | 91 | 188 |
| Parotid_R | 99 | 92 | 191 |
| SpinalCord | 104 | 103 | 207 |
