# Supplementary material for: A general model for head and neck auto‐segmentation with patient pre‐treatment imaging during adaptive radiation therapy
Source: Med Phys. 2025 Mar 7;52(6):4590–7. doi: 10.1002/mp.17732 (PMC12149676; doi:10.1002/mp.17732)
Supplement: Supplementary file 4 — Supplementary Table 2: Mean volumes (cm3) for the GTVp and OARs in the PMCC‐REPLAN dataset (110 patients, 220 CT images) split by pre‐ and mid‐treatment images. Significant differences (*) in volume between pre‐ and mid‐treatment images were calculated using a paired t‐test (p < 0.05). [file MP-52-4590-s002.docx]

| Structure | Volume (cm^3^) | | Volume diff. (cm^3^) | Volume diff. (%) |
| --- | --- | --- | --- | --- |
|  | Pre-treatment | Mid-treatment |  |  |
| Bone_Mandible | 77.48 | 77.25 | -0.23 | -0.30 |
| BrachialPlex_L | 9.08 | 8.56 | * -0.52 | * -5.77 |
| BrachialPlex_R | 8.56 | 8.57 | 0.01 | 0.10 |
| Brain | 1384.14 | 1403.99 | * 19.85 | * 1.43 |
| Brainstem | 26.20 | 26.49 | 0.29 | 1.09 |
| Cavity_Oral | 113.77 | 114.87 | 1.10 | 0.97 |
| Esophagus_S | 11.58 | 12.61 | * 1.02 | * 8.84 |
| GTVp | 35.53 | 32.45 | -3.08 | -8.66 |
| Glnd_Submand_L | 10.97 | 11.12 | 0.14 | 1.32 |
| Glnd_Submand_R | 10.76 | 10.87 | 0.11 | 1.03 |
| Larynx | 42.44 | 43.12 | 0.67 | 1.59 |
| Lens_L | 0.30 | 0.32 | 0.02 | 5.82 |
| Lens_R | 0.31 | 0.32 | 0.01 | 1.86 |
| Musc_Constrict | 23.31 | 23.63 | 0.32 | 1.37 |
| Parotid_L | 34.08 | 31.37 | * -2.71 | * -7.96 |
| Parotid_R | 35.09 | 32.75 | * -2.34 | * -6.66 |
| SpinalCord | 27.89 | 27.88 | -0.01 | -0.04 |
