# Supplementary material for: A general model for head and neck auto‐segmentation with patient pre‐treatment imaging during adaptive radiation therapy
Source: Med Phys. 2025 Mar 7;52(6):4590–7. doi: 10.1002/mp.17732 (PMC12149676; doi:10.1002/mp.17732)
Supplement: Supplementary file 5 — Supplementary Table 3: Patient demographics for PMCC‐REPLAN dataset. [file MP-52-4590-s007.docx]

| Demographic | | Values |
| --- | --- | --- |
| Age [years] | Median | 62 |
|  | Range | 20 - 85 |
| Dose (before mid-treatment imaging) [gray] | Median | 2000 |
|  | Range | 300 - 4600 |
| Dose (total) [gray] | Median | 7000 |
|  | Range | 4200 - 7000 |
| Primary tumor site | Unknown | 25 (21%) |
|  | Tongue | 19 (16%) |
|  | Tonsil | 17 (14%) |
|  | Oropharynx | 16 (13%) |
|  | Skin | 10 (8%) |
|  | Larynx | 8 (7%) |
|  | Nasopharynx | 7 (6%) |
|  | Hypopharynx | 5 (4%) |
|  | Oral cavity | 5 (4%) |
|  | Glottis | 3 (3%) |
|  | Maxilla | 2 (2%) |
|  | Mandible | 1 (1%) |
|  | Paranasal sinus | 1 (1%) |
| Sex | Female | 17 (15%) |
|  | Male | 93 (84%) |
| Time between pre/mid-treatment imaging [days] | Median | 36 |
|  | Range | 14 - 79 |
