# Supplementary material for: A general model for head and neck auto‐segmentation with patient pre‐treatment imaging during adaptive radiation therapy
Source: Med Phys. 2025 Mar 7;52(6):4590–7. doi: 10.1002/mp.17732 (PMC12149676; doi:10.1002/mp.17732)
Supplement: Supplementary file 6 — Supplementary Table 4: OARs that failed to converge (non‐zero validation DSC) for a given model and test fold. [file MP-52-4590-s004.docx]

| Model | Test folds | Non-converged OAR |
| --- | --- | --- |
| Reference | 0 | Brainstem |
|  | 3 | Glnd_Submand_L |
|  | 2 | Lens_R |
| Patient-specific | 2, 3 | Lens_L |
|  | 1, 4 | Lens_R |
| General adaptive | 0 | BrachialPlex_R |
|  | 1, 2, 3, 4 | Lens_L |
