# Supplementary material for: A general model for head and neck auto‐segmentation with patient pre‐treatment imaging during adaptive radiation therapy
Source: Med Phys. 2025 Mar 7;52(6):4590–7. doi: 10.1002/mp.17732 (PMC12149676; doi:10.1002/mp.17732)
Supplement: Supplementary file 7 — Supplementary Table 5: MSD (mean ± std. deviation) for reference (RM), patient‐specific (PSM), general adaptive (GAM), and rigid image registration (RIR) auto‐segmentation methods, trained and evaluated on the PMCC‐REPLAN dataset. Mean DSC is averaged over the five test folds for the GTVp and 16 OARs, with rows ordered by largest difference between GAM and RM methods. Significant improvements (p < 0.05) in GAM performance over RM (*) and PSM (†) methods are shown, in addition to significant differences between GAM and RR (‡). No std. deviation was available for the GAM for the left lens due to convergence for a single test fold only. [file MP-52-4590-s005.docx]

| Structure | MSD [mm] | | | |
| --- | --- | --- | --- | --- |
|  | Reference | Patient-specific | General adaptive | Rigid registration |
| GTVp | 20.01 ± 2.42 | 12.41 ± 5.52 | * 4.33 ± 2.12 | 3.25 ± 0.81 |
| BrachialPlex_L | 4.74 ± 1.01 | 3.84 ± 0.68 | 2.93 ± 0.86 | 2.95 ± 0.78 |
| BrachialPlex_R | 4.93 ± 0.52 | 3.74 ± 0.94 | *† 2.39 ± 0.40 | 2.87 ± 1.03 |
| Larynx | 4.14 ± 0.84 | 3.79 ± 1.63 | 2.51 ± 0.33 | 2.68 ± 0.50 |
| Esophagus_S | 3.95 ± 0.88 | 3.25 ± 0.90 | *† 2.71 ± 1.10 | 3.21 ± 0.76 |
| Glnd_Submand_L | 2.17 ± 0.10 | 2.13 ± 0.74 | 1.73 ± 0.28 | 2.05 ± 0.58 |
| Glnd_Submand_R | 2.38 ± 0.34 | 2.46 ± 0.28 | 2.21 ± 0.34 | 2.38 ± 0.38 |
| Parotid_L | 3.50 ± 2.23 | 2.43 ± 0.15 | 1.98 ± 0.23 | 2.84 ± 0.70 |
| Parotid_R | 2.50 ± 0.11 | 2.35 ± 0.20 | * 2.08 ± 0.22 | 2.81 ± 0.99 |
| Cavity_Oral | 3.44 ± 0.11 | 2.88 ± 0.31 | * 2.53 ± 0.33 | 2.30 ± 0.37 |
| Musc_Constrict | 3.60 ± 0.51 | 3.48 ± 1.38 | 2.56 ± 0.54 | 3.01 ± 0.84 |
| Brainstem | 2.17 ± 0.17 | 2.13 ± 0.20 | *† 1.74 ± 0.13 | 2.76 ± 0.99 |
| Bone_Mandible | 1.13 ± 0.34 | 1.13 ± 0.23 | ‡ 0.98 ± 0.07 | 2.47 ± 0.45 |
| Brain | 1.62 ± 0.52 | 1.57 ± 0.47 | 1.51 ± 0.20 | 2.36 ± 1.04 |
| SpinalCord | 1.58 ± 0.43 | 1.69 ± 0.27 | ‡ 1.46 ± 0.12 | 2.98 ± 0.61 |
| Lens_L | 1.41 ± 0.24 | 1.04 ± 0.34 | 1.82 | 4.29 ± 2.23 |
| Lens_R | 1.43 ± 0.51 | 1.85 ± 0.26 | 1.76 ± 0.89 | 4.04 ± 2.09 |
| All structures | 3.80 ± 4.22 | 3.07 ± 2.49 | 2.19 ± 0.73 | 2.90 ± 0.56 |
