# Supplementary material for: A general model for head and neck auto‐segmentation with patient pre‐treatment imaging during adaptive radiation therapy
Source: Med Phys. 2025 Mar 7;52(6):4590–7. doi: 10.1002/mp.17732 (PMC12149676; doi:10.1002/mp.17732)
Supplement: Supplementary file 8 — Supplementary Table 6: DSC (mean ± std. deviation) and MSD (mean ± std. deviation) for reference and nnU‐Net models trained and evaluated on the PMCC‐REPLAN dataset. Mean DSC and MSD are averaged over all test folds for the GTVp and 16 OARs. Significant improvements in performance (p < 0.05) are shown for reference (*) and nnU‐Net (†) models. [file MP-52-4590-s001.docx]

| Structure | DSC | | MSD [mm] | |
| --- | --- | --- | --- | --- |
|  | Reference | nnU-Net | Reference | nnU-Net |
| Bone_Mandible | 0.87 ± 0.03 | 0.91 ± 0.01 | 1.13 ± 0.34 | 0.75 ± 0.14 |
| BrachialPlex_L | 0.38 ± 0.03 | 0.37 ± 0.03 | 4.74 ± 1.01 | 4.35 ± 0.76 |
| BrachialPlex_R | 0.35 ± 0.03 | 0.39 ± 0.04 | 4.93 ± 0.52 | 4.22 ± 0.95 |
| Brain | 0.95 ± 0.02 | 0.97 ± 0.00 | 1.62 ± 0.52 | 1.17 ± 0.13 |
| Brainstem | 0.79 ± 0.02 | 0.83 ± 0.01 | 2.17 ± 0.17 | 1.78 ± 0.15 |
| Cavity_Oral | 0.80 ± 0.01 | 0.81 ± 0.02 | 3.44 ± 0.11 | 3.26 ± 0.39 |
| Esophagus_S | 0.57 ± 0.03 | † 0.63 ± 0.03 | 3.95 ± 0.88 | 3.94 ± 0.79 |
| GTVp | 0.17 ± 0.02 | 0.18 ± 0.06 | 20.01 ± 2.42 | 16.59 ± 5.81 |
| Glnd_Submand_L | 0.71 ± 0.03 | 0.74 ± 0.05 | 2.17 ± 0.10 | 1.87 ± 0.34 |
| Glnd_Submand_R | 0.68 ± 0.03 | † 0.76 ± 0.01 | 2.38 ± 0.34 | † 1.68 ± 0.15 |
| Larynx | 0.68 ± 0.04 | 0.73 ± 0.03 | 4.14 ± 0.84 | 3.88 ± 1.10 |
| Lens_L | 0.50 ± 0.08 | 0.36 ± 0.34 | 1.41 ± 0.24 | 0.98 ± 0.10 |
| Lens_R | 0.50 ± 0.15 | 0.30 ± 0.28 | 1.43 ± 0.51 | 1.92 ± 1.21 |
| Musc_Constrict | 0.54 ± 0.02 | 0.56 ± 0.03 | 3.60 ± 0.51 | 2.92 ± 0.57 |
| Parotid_L | 0.74 ± 0.04 | 0.78 ± 0.03 | 3.50 ± 2.23 | 2.12 ± 0.23 |
| Parotid_R | 0.76 ± 0.01 | † 0.80 ± 0.02 | 2.50 ± 0.11 | † 1.96 ± 0.17 |
| SpinalCord | 0.76 ± 0.02 | 0.77 ± 0.04 | 1.58 ± 0.43 | 1.38 ± 0.21 |
| All structures | 0.63 ± 0.20 | 0.64 ± 0.24 | 3.81 ± 4.10 | 3.22 ± 3.64 |
